# Frontline Febrile Neutropenia Antibiotic Treatment Across Institution Types: A Multicenter Survey Study

**DOI:** 10.1093/ofid/ofag180

**Published:** 2026-04-02

**Authors:** Maia Castillo, Derick Gross, Stephanie J Harding

**Affiliations:** Department of Pharmacy, Oregon Health & Science University, Portland, Oregon, USA; Department of Pharmacy, Wesley Medical Center, Wichita, Kansas, USA; Department of Pharmacy, Wesley Medical Center, Wichita, Kansas, USA

**Keywords:** febrile neutropenia, stewardship

## Abstract

The inherently variable nature of febrile neutropenia contributes to inconsistent adherence to Infectious Diseases Society of America/American Society of Clinical Oncology guidelines. This survey collected information on how institutions treat febrile neutropenia. Study responses showed that institutions with internal treatment protocols also reported increased adherence to guidelines, which is an accessible intervention for most institutions to implement.

## Teaser

In a multicenter survey analyzing the differences in febrile neutropenia management based on institution types, responders from institutions with internally published treatment protocols had lower perceived utilization of agents for anti-MRSA coverage. Larger institutions and academic medical centers were more likely to have increased specialist presence from infectious diseases and oncology.

Febrile neutropenia (FN) is a common sequela of cancer with approximately 30% of patients receiving chemotherapy for solid tumors and 80% of patients receiving chemotherapy for leukemia experiencing FN at some point during treatment [1]. FN can result in serious morbidity and mortality, with a recent study reporting up to an 18.24% mortality rate, and a 2012 study reporting a mean hospital stay of 9.6 days with a mean cost of $24 770 per episode—episodes that may lead to delays in oncology treatment or dose reductions [2, 3]. Furthermore, fever etiology is determined in less than half of FN episodes with only about a third having documented bacteremia [1]. Even with the uncertain etiology, the risk of infection and morbidity in neutropenic patients necessitates empiric antipseudomonal antibiotic therapy within 1 hour [4]. The most common causes of bacteremia in FN are Enterobacteriaceae and *Pseudomonas*; however, gram-positive bacteria have become more prevalent in recent years [1, 5]. Even with this increase, the American Society of Clinical Oncology guidelines only indicate gram-positive coverage in certain circumstances, and highlight the importance of covering gram-negative pathogens [1].

Guidelines on FN treatment have been in place for years, but little is known about adherence to the guidelines by providers, with some previous smaller surveys suggesting a wide variety in practice. FN treatment is inherently variable, which contributes to the inconsistency in guideline adherence. Deviations from guideline treatment can be detrimental, with a 2013 study finding that guideline-recommended antibiotics reduced mortality by 37% [6]. The guidelines emphasize gram-positive coverage only in certain situations, and despite a 2017 review of previous randomized controlled trials showing no mortality benefit with empiric gram-positive coverage, a separate study found a 37.7% increase in frontline vancomycin usage in FN from 2000 to 2010 [6]. Due to this seeming contradiction with the guidelines, our survey aimed to gain information on how institutions treat FN and determine if any patterns emerged between different institution or provider types.

## METHODS

The survey tool Qualtrics was used to create a 12-question survey to collect data including respondent's job title, size and type of institution, and service line responsible for FN treatment both initially, when a patient is first admitted, and primarily, during a patient's hospital course. Presence of formal protocol for FN treatment, as well as percentage of patients who receive empiric methicillin-resistant *Staphylococcus aureus* (MRSA) and pseudomonal coverage was included in the survey. Respondents were also asked if their institution regularly used double antipseudomonal coverage, and if so, with what agents. Study questions were created and agreed upon by all investigators prior to survey publication. The purpose of the study was to analyze differences in FN management based on institution type. Subgroups for analysis were determined a priori through predetermined survey answer choices as described in Table 1. Certain subgroups were grouped for analysis post hoc due to response rate as delineated in Table 2.

**Table 1.** Practice Site Demographics and Clinical Practices (N = 84)

Characteristic	Demographics	Responses, No. (%)
Size of institution (No. of beds)	50–100	4 (4.8)
	101–300	13 (15.5)
	301–500	19 (22.6)
	501–700	15 (17.9)
	>700	32 (38.1)
	No response	1 (1.2)
Site classification	Academic medical center	53 (63.1)
	Community teaching hospital	21 (25.0)
	Community non-teaching hospital	7 (8.3)
	Other	3 (3.6)
Presence of formal protocol for febrile neutropenia treatment	Yes	51 (60.7)
	No	33 (39.3)
Service line initially responsible for treatment	Hospitalist	23 (27.4)
	Infectious diseases	3 (3.6)
	Oncology	34 (40.5)
	Emergency department	22 (26.2)
	NA	2 (2.4)
Service line primarily responsible for treatment	Hospitalist	14 (16.7)
	Infectious diseases	23 (27.4)
	Oncology	47 (56.0)

Abbreviation: NA, not applicable.

**Table 2.** Subgroup Analyses

Comparator	AMC	Non-AMC	*P* Value	>500 Beds	≤500 Beds	*P* Value	Protocol	No Protocol	*P* Value
	n = 53	n = 31		n = 47	n = 36		n = 51	n = 33	
Internal protocol for treatment of febrile neutropenia published	36 (67.9)	15 (48.4)	.077	31 (66.0)	19 (52.8)	.224	51 (100)	0 (0)	
Service line initially responsible									
Emergency	14 (26.4)	8 (25.8)	.958	12 (25.5)	10 (27.8)	.844	15 (29.4)	7 (21.2)	.473
Hospitalist	10 (18.9)	13 (41.9)	.051	7 (14.9)	16 (44.4)	**.011**	12 (23.5)	11 (33.3)	.402
Infectious diseases	1 (1.9)	2 (6.5)	.285	2 (4.3)	1 (2.8)	.726	2 (3.9)	1 (3.0)	.832
Oncology	27 (50.9)	7 (22.6)	**.047**	25 (53.2)	9 (25.0)	**.047**	20 (39.2)	14 (42.4)	.821
Unspecified	1 (1.9)	1 (3.2)	.701	1 (2.1)	0 (0)	.381	2 (3.9)	0 (0)	.255
Service line primarily responsible									
Hospitalist	5 (9.4)	9 (29.0)	**.034**	5 (10.6)	9 (25.0)	.114	8 (15.7)	6 (18.2)	.784
Infectious diseases	13 (24.5)	10 (32.3)	.513	9 (19.1)	14 (38.9)	.09	14 (27.5)	9 (27.3)	.987
Oncology	35 (66.0)	12 (38.7)	.106	33 (70.2)	13 (36.1)	**.039**	29 (56.9)	18 (54.5)	.89
Perceived anti-MRSA treatment utilization									
Appropriate	28 (52.8)	10 (32.3)	.176	24 (51.1)	14 (38.9)	.417	24 (47.1)	14 (42.4)	.758
Overutilized	20 (37.7)	17 (54.8)	.254	19 (40.4)	18 (50.0)	.517	20 (39.2)	17 (51.5)	.407
Underutilized	1 (1.9)	0 (0)	.444	1 (2.1)	0 (0)	.381	1 (2.0)	0 (0)	.421
Unspecified	4 (7.5)	4 (12.9)	.443	3 (6.4)	4 (11.1)	.462	6 (11.8)	2 (6.1)	.408

Data are presented as No. (%) unless otherwise indicated. Bolded values indicate *P*-values less than 0.05.

Abbreviations: AMC, academic medical center; MRSA, methicillin-resistant *Staphylococcus aureus*.

The survey was distributed via email to listservs: the American College of Clinical Pharmacy (ACCP) Infection Diseases Practice and Research Network, the ACCP Hematology/Oncology Practice and Research Network, and the IDSA. All listservs include members who have signed up and consented to receive emails such as this survey, and completion of the survey was voluntary. The platform Qualtrics was also used to collect responses, and no personal identifying information was gathered or saved.

Descriptive statistics were used for all demographic qualitative data and to summarize survey responses. Chi-squared tests were used for comparing results between the post hoc aggregated subgroups listed in Table 2. Mann–Whitney *U* tests, and Kruskal–Wallis tests were used to analyze continuous data between those subgroups as indicated depending on the number of subgroups being analyzed.

## RESULTS

A total of 89 survey responses were received, 5 of which were excluded as they only included demographic information; therefore, 84 responses were included in the analysis. Demographic information for all the responses was broken down by bed size and site classification (Table 1). The largest percentage of responders were from institutions with >700 beds (38.1%). For site classification, more than half of responders were from academic medical centers (AMCs) (63.1%). Of note, 3 responses indicated “other” as site classification and the free text responses indicated a private community hematology/oncology clinic, cancer center, or a private children's oncology hospital. More than half of responders (61%) indicated that their institution did have an internally published protocol for treating FN. Oncology as a service line had the largest percentage for both initial and primary responsibility for treatment decisions, with 40.5% and 56.0%, respectively.

When asked about empiric antipseudomonal use, a total of 62 responders indicated that most of their patients received coverage, with a median of 100% and an interquartile range spanning from 99.3% to 100%. There were a total of 72 responders who indicated if their institution routinely used 2 antipseudomonal agents, with 65% (47) responding “never,” 33% (24) responding “some of the time,” <1% (1) responding “half of the time,” and 4% (3) responding “most of the time.” Additionally, a total of 70 respondents indicated a specific percentage of patients who routinely received anti-MRSA coverage, with the median being 30% of patients, and the interquartile range spanning from 25% to 83%. For perception, 50% (38 of 76) of responders indicated they felt empiric anti-MRSA usage at their institution was appropriate, 49% (37 of 76) indicated they felt anti-MRSA coverage was overutilized, and 1 respondent indicated they felt anti-MRSA treatment was underutilized at their institution.

When comparing AMCs to non-AMCs (Table 2), there was more initial oncology responsibility for treatment (50.9% vs 22.6%; *P* = .047) and less empiric MRSA coverage (*P* = .039). When comparing institutions with >500 beds to those with ≤500 beds (Table 2), similar results were seen, with more initial oncology responsibility in larger locations (53.2% vs 25%; *P* = .047), more primary oncology responsibility in larger locations (70.2% vs 36.1%; *P* = .039), and less empiric MRSA coverage (*P* = .02). When comparing institutions with internal protocols versus without (Table 2), there was less empiric MRSA coverage with a protocol (*P* = .006). No statistically significant differences in percentage of patients treated with MRSA or pseudomonal coverage were found when comparing service lines responsible for FN treatment.

## DISCUSSION

The survey responses indicated that institutions with guidelines or protocols for FN treatment reported lower percentages of patients treated with MRSA coverage, highlighting the need for available guidance and education on how to treat these cases. Of note, the survey did not ask respondents to provide details on the form of their institution's protocol, which could be implemented in several different forms. Information on National Cancer Institute (NCI) designation of institutions was not collected in this survey, notable as previous survey data of NCI-designated cancer centers found that 94% of responses indicated presence of FN guidelines, 90% of which recommended against routine empiric gram-positive coverage [7].

Additionally, there were no responses from critical access hospitals or institutions with <50 beds, underscoring the bias toward larger AMCs. This bias could be partially explained due to the survey being sent through IDSA and ACCP listservs where specialists may be overrepresented. Despite this bias toward larger AMCs, responses were obtained from a variety of infectious diseases and oncology professionals. Physicians and pharmacists responded, as well as residents from pharmacy, medical fellows, and even academic professionals. Having this breadth of responses allows for a larger pool of viewpoints for this perception-based survey.

Though respondents from larger institutions and AMCs reported more oncology involvement and a lower reported percentage of empiric anti-MRSA coverage for FN patients, there was no statistically significant difference in perception of utilization between any subgroups. Using perception-based data for both reported percentage of use and perception of utilization may have caused an issue. There are situations where empiric MRSA coverage would be warranted, included in the guidelines, which may skew perception data as anti-MRSA coverage utilization could have been indicated [1]. The lack of significant difference in perceived utilization could also have reflected who the survey was sent to, with infectious diseases and oncology specialists possibly having similar perceptions on anti-MRSA coverage used empirically for FN.

Overall treatment practices across the sites from respondents largely concur with current guidelines. Nearly all respondents indicated that all FN patients received antipseudomonal therapy empirically, and the median reported number of patients who received empiric anti-MRSA treatment was only 30%. Differences were primarily seen in the larger institutions and AMCs where there may be more access to specialists; however, there was no significant difference in anti-MRSA use between specific service lines responsible either initially or primarily.

Respondents from institutions with internally published guidelines for FN management also indicated an increase in guideline adherence, which is an easy modification an institution can implement. Having a small sample size with perception-based data was a large limitation of this study; therefore, future studies with larger sample sizes and concrete usage data could be pursued to better understand FN treatment guideline adherence.
